# Understanding the utilization of primary health care services by Indigenous men: a systematic review

**DOI:** 10.1186/s12889-018-6093-2

**Published:** 2018-10-23

**Authors:** Kootsy Canuto, Alex Brown, Gary Wittert, Stephen Harfield

**Affiliations:** 1 Wardliparingga Aboriginal Research Unit, South Australian Health and Medical Research Institute, Adelaide, Australia; 2 Freemasons Foundation Centre for Men’s Health, University of Adelaide, Adelaide, Australia; 3 Centre of Research Excellence in Aboriginal Chronic Disease Knowledge Translation and Exchange (CREATE), University of Adelaide, Adelaide, Australia; 4 Sansom Institute for Health Research, University of South Australia, Adelaide, Australia

**Keywords:** Indigenous, Aboriginal, Torres Strait islander, Men, Utilization, Primary health care

## Abstract

**Background:**

Aboriginal and Torres Strait Islander men experience worse health outcomes and are the most marginalized and disadvantaged population group in Australia. Primary health care services are critical to providing both clinical and social and emotional support, however, remain underutilized by Aboriginal and Torres Strait Islander men. This review aims to better understand the utilization of primary health care services by Indigenous men and assess the effectiveness of strategies implemented to improve utilization.

**Methods:**

A four-step search strategy was employed across four databases to find peer-reviewed publications and grey literature from Australia, New Zealand, Canada and America. The search began in March 2015 and included the following databases PubMed, CINAHL, Informit (Indigenous collection) and Embase. Additional databases and websites were also searched for grey literature, reference lists of included publications were searched for additional studies and relevant experts were consulted.

**Results:**

The literature search found seven articles that met the inclusion criteria; four describing three research projects, plus three expert opinion pieces. The search was unable to find published research on strategies implemented to improve primary health care utilization by Indigenous men.

There is limited published research focused on the utilization of primary health care by Indigenous men. From the identified papers Indigenous men described factors impacting utilization which were categorized into three primary organizing themes; those related to health services, the attitudes of Indigenous men and knowledge. It is evident from the identified papers that improvements in Indigenous health can only occur if future programs are developed in collaboration with health services and Indigenous men to address differing requirements.

**Conclusions:**

Currently, health systems in Australia are limited in their ability to improve the health and wellbeing of Aboriginal and Torres Strait Islander males without such strategies. Future research should focus on evaluating the implementation of men specific utilization strategies. It is through evidence-based research that subsequent policies and programs can be made and implemented to improve Indigenous men’s health.

## Background

Like other Indigenous peoples from colonized countries including New Zealand, Canada and the Unites States of America, Aboriginal and Torres Strait Islander people experience worse health outcomes than their non-Indigenous counterparts [1]. Although there have been improvements in the life expectancy among Australian males in the last 30 years, there has been little improvement noted in the life expectancy gap between Aboriginal and Torres Strait Islander men and their non-Indigenous counterparts, currently estimated to be 10.6 years [2, 3]. Although there are issues with comparing data across countries [4], the data clearly shows life expectancy gaps between Indigenous peoples from Australia, New Zealand, Canada and America and their non-Indigenous counterparts [5].

Against virtually all markers of health and social status, across the life span, Aboriginal and Torres Strait Islander people are the most marginalized and disadvantaged in Australian society [6]. Internationally, the health disparities of Aboriginal and Torres Strait Islander people “are as large as those seen in any other high-income country”[7] (p. 1). Aboriginal and Torres Strait Islander men are at a particularly elevated risk of psychological illness, drug and alcohol issues, engagement with the justice system, suicide and self-harm [8–10]. These issues not only contribute to poor quality of life, but contribute to the onset and severity of a range of health conditions, including cardiovascular and metabolic diseases [9].

Adverse historical and contemporary traumas experienced by Aboriginal and Torres Strait Islander men have been directly linked to their current adverse health profiles [11, 12]. The enduring sociocultural effects of colonization continues to have a detrimental effect on the health and social and emotional wellbeing of Indigenous people [13–19]. Prior to colonization Indigenous men and women “had defined roles according to age and gender”[19] (p.5). Colonization significantly altered the role of the Indigenous man [8, 20–23]. Traditional power and authority that Indigenous men had was often taken from them as a consequence of direct conflict and government policy, with significant impacts on specific roles as family protectors and providers for their community [22].

Aboriginal and Torres Strait Islander males are constantly being portrayed by perpetuating negative stereotypes such as being lazy, always drunk, are violent, uneducated, primitive, and being problems, all of which can hinder improvements to their health status and has led to the development of health and social policies that continue to suggest that these men are responsible a range of issues facing Indigenous families and communities. Consequently, health disparity is “being seen as a result of Aboriginal people’s own failings. Worse still, they are being actively marginalized from influencing any path to potential solutions”[24] (p.97).

Primary health care services are critical to providing both clinical and social and emotional support for Aboriginal and Torres Strait Islander people. Brown suggests that “improving access to primary care stands as a critical target for improving health status among Indigenous Australians”[25] (p.815). However, primary health care services remain underutilized by Aboriginal and Torres Strait Islander men [26–28]. The reasons for this are not well documented or described and are likely to be complex. Current health system approaches fail to acknowledge that Aboriginal and Torres Strait Islander men have requirements for accessing and utilizing health systems that are different to Aboriginal and Torres Strait Islander women and their non-Indigenous counterparts [24]. Strategies are required to improve access and ultimately health outcomes among Aboriginal and Torres Strait islander men.

In Australia, health systems need to acknowledge the needs of all of their clients if the health and wellbeing of all Australians is to improve. However, such a health system cannot exist “without consideration of the ways in which culture intersects with issues of poverty and equity, including access and utilization of health care, individual and institutional racism, and a lack of cultural competence on the part of health providers and programs”[17] (p.2). Unfortunately, little action has been taken in this regard. The reorienting of services to decrease or remove barriers to care, and ensuring that the services are acceptable, of high quality and sensitive to the needs and demands of Aboriginal and Torres Strait Islander men remains a difficult task [25].

To meet the needs of Aboriginal and Torres Strait Islander people, Aboriginal Community Controlled Health Services (ACCHS) have been established [29]. There are 143 ACCHS in Australia, providing comprehensive primary health care services to local Aboriginal and Torres Strait Islander people [29]. The ACCHS are controlled by local community representatives and ‘represent the only truly effective and culturally valid mode of delivering effective and sustainable primary health care services to Aboriginal Peoples’ [29]. Similar comprehensive Indigenous primary health care services have also been established in New Zealand, Canada and America [30].

This review aims to (i) better understand the utilization of primary health care services by Indigenous men, and (ii) assess the effectiveness of strategies aimed at increasing the utilization of primary health care services by Indigenous men. This review had two research questions: 1) What factors impact the utilization of primary health care services by Indigenous men? 2) What strategies have been implemented to increase utilization of primary health care services by Indigenous men and how effective were they?

## Methods

Prior to commencing the review, a search of the Joanna Briggs Database of Systematic Reviews and Implementation Reports, the Cochrane Library, CINAHL, PubMed and PROSPERO revealed that no systematic review (either published or underway) has been conducted on this topic.

The authors developed and published a review protocol for this comprehensive systematic review [31]. This comprehensive literature review was guided by the Joanna Briggs Institute guidelines for systematic review and synthesis of qualitative data [32]. Studies from New Zealand, Canada and America were also included in the review due to the countries shared experience with colonization and their high disparity of health and wellbeing between the Indigenous and non-Indigenous peoples within each country.

### Inclusion criteria

Each research question had their own inclusion criteria. Only the criteria related to the types of participants/population were the same for both searches.

*Question 1: What factors impact the utilization of primary health care services by Indigenous men?*

### Types of participants/population

Papers will be included if most of the participants in the study are men (aged 18 years and older) and are Indigenous to Australia (Aboriginal and/or Torres Strait Islander), New Zealand (Maori), Canada (First Nations) and America (native American).

### Phenomena of interest

Studies that investigate the experience of clients with primary health care services will be included.

### Context

Qualitative studies that explore client views or experiences relating to barriers and enablers to access or their experience with primary health care services will be included.

### Types of studies

Qualitative studies to be included will be descriptive, ethnography, phenomenology and grounded theory studies, action research and evaluations, including developmental evaluations.

Published expert opinion will also be considered for inclusion.

*Question 2: 2) What strategies have been implemented to increase utilization of primary health care services by Indigenous men and how effective were they?*

### Types of participants/population

Papers will be included if most of the participants in the study are men (aged 18 years and older) and are Indigenous to Australia (Aboriginal and/or Torres Strait Islander), New Zealand (Maori), Canada (First Nations) and America (native American).

### Types of intervention

The review will consider studies on services that implement strategies or programs to increase health service utilization by Indigenous men:

Quantitative component: studies that evaluate health service utilization/access.

Qualitative component: studies that investigate client views or experiences related to these strategies.

### Context

The review will consider studies whose context is primary health care services.

Acute care, chronic disease management, tertiary care or short-term rehabilitation clinics will not be considered.

### Comparator

The quantitative component of this study will consider studies that evaluate and investigate primary health care services that implement a strategy to increase service utilization by Indigenous men. This may be a group of men who received the strategy compared to a group of men who did not receive the strategy or a study that compares services that did and did not receive the strategy. This review will also consider studies that have no comparator.

### Types of outcomes

The quantitative component of this question will consider studies that include, but not limited to, the following outcome measures; occasions of care and client numbers.

### Types of studies

Quantitative studies to be included are randomized controlled trials, non-randomized controlled trials, economic evaluations and costing studies (including model-based studies), retrospective and prospective cohort studies, case control studies, health service studies, health service evaluations, analytic cross-sectional studies and descriptive epidemiological study designs.

Qualitative studies to be included will be descriptive, ethnography, phenomenology and grounded theory studies, action research and evaluations, including developmental evaluations.

Mixed methods studies will also be considered for this review.

Papers were included if they met the inclusion criteria for one of the review questions. Qualitative papers that had explored client views or experiences related to barriers and enablers to access or their experience with primary health care services were included for the first review question. Papers that described the implementation of strategies or programs that aimed to increase health service utilization by Indigenous men were included to answer review question two.

For this review a primary health care center was defined as a health service outside an inpatient setting where patients can directly access, such as general practices, outpatient treatment and allied health services. Studies that were based in an acute care setting, or were focused on participants with a chronic condition, tertiary care or rehabilitation clinic were not included. Mixed methods publications that included a qualitative component which met the inclusion criteria were also included.

Expert opinion publications were reviewed if they discussed/offered opinions to the barriers and enablers for Aboriginal and Torres Strait Islander men utilizing primary health care services and were authored by Aboriginal and Torres Strait Islander men. Advice from male Aboriginal and Torres Strait Islander health research experts was sought to confirm the papers author(s) were indeed considered experts in the field and were Aboriginal and/or Torres Strait Islander men. These expert opinion papers were assessed for quality and contributed to the discussion of the paper, however, as they are not research studies, they are not described in the results section of this review.

### Search strategy

A four-step search strategy was employed to find peer-reviewed publications and grey literature. Key words (listed in the review protocol) were used to search PubMed to identify additional keywords and index terms [32]. A search was then undertaken across four databases; PubMed, CINAHL, Informit (Indigenous collection) and Embase. The following databases and websites were also searched for grey literature; ProQuest, Trove, the National Aboriginal Community Controlled Health Organisation website, Australian Indigenous Health*Info*Net, National Library of Australia and the Lowitja Institute. The reference list of included publications was searched for additional studies. In addition, relevant experts were also asked if they were aware of additional missing studies. The final two steps did not result in additional papers being included.

The search strategy in PubMed was as follows, this was modified as required in the other databases: Indigenous[tiab] OR Aborigin*[tiab] OR Torres Strait Islander[tiab] OR Inuit[tiab] OR Maori[tiab] OR American Indian[tiab] OR Native American[tiab] OR First Nation[tiab] OR Oceanic Ancestry Group[Mesh] OR “American Native Continental Ancestry Group”[Mesh] AND strateg* OR utilis* OR access* OR approach* OR tactic* OR engag* OR intervent* OR program* AND Primary health[tiab] OR primary care[tiab] OR “Health Care Quality, Access, and Evaluation”[Mesh] OR “Primary health care”[Mesh] OR “Health Services, Indigenous”[Mesh]. The search of data bases was conducted on the 15th of March 2015, was restricted to those published in English, with no date restrictions.

The lead author, KC, identified articles that appeared to meet the inclusion criteria from their title and abstract. The full text of these articles was retrieved and reviewed by KC and SH to confirm if they met the inclusion criteria.

### Assessment of methodological quality

The methodological quality of included studies was assessed by KC and SH using standardized critical appraisal instruments. The Joanna Briggs Institute (JBI) have produced a suite of critical appraisal tools to assess the quality of publications for the purposes of systematic review. The suite includes tools designed for the review of different types of publications. Qualitative papers (or qualitative components of mixed methods papers) were assessed using the JBI Qualitative Assessment and Review Instrument (JBI-QARI) [32]. Textual papers were assessed using the JBI Narrative, Opinion and Text Assessment and Review Instrument (JBI-NOTARI) [32]. When the reviewers had differing opinions on an article’s quality, it was resolved by discussion and if needed a third party was consulted.

### Data extraction

Data extraction was assisted using standardized tools. JBI-QARI for qualitative data, and JBI-NOTARI for textual data [32]. Two reviewers each completed data extraction on the included papers, then cross-checked each for completion and accuracy.

### Data synthesis

The articles that met the inclusion criteria for research question one was read several times over to extract the findings related to participants experiences with primary health care services. Information was extracted from the results and discussion sections of the papers. The factors impacting the three studies were combined as were findings that were the same or similar. The barriers and enablers for Indigenous men accessing primary health care services were grouped into organizing themes (Table 4). Data synthesis was not conducted on the expert opinion manuscripts, as they did not describe qualitative findings from a study, however, the manuscripts have contributed to the discussion.

## Results

### Study selection

**Fig. 1:**
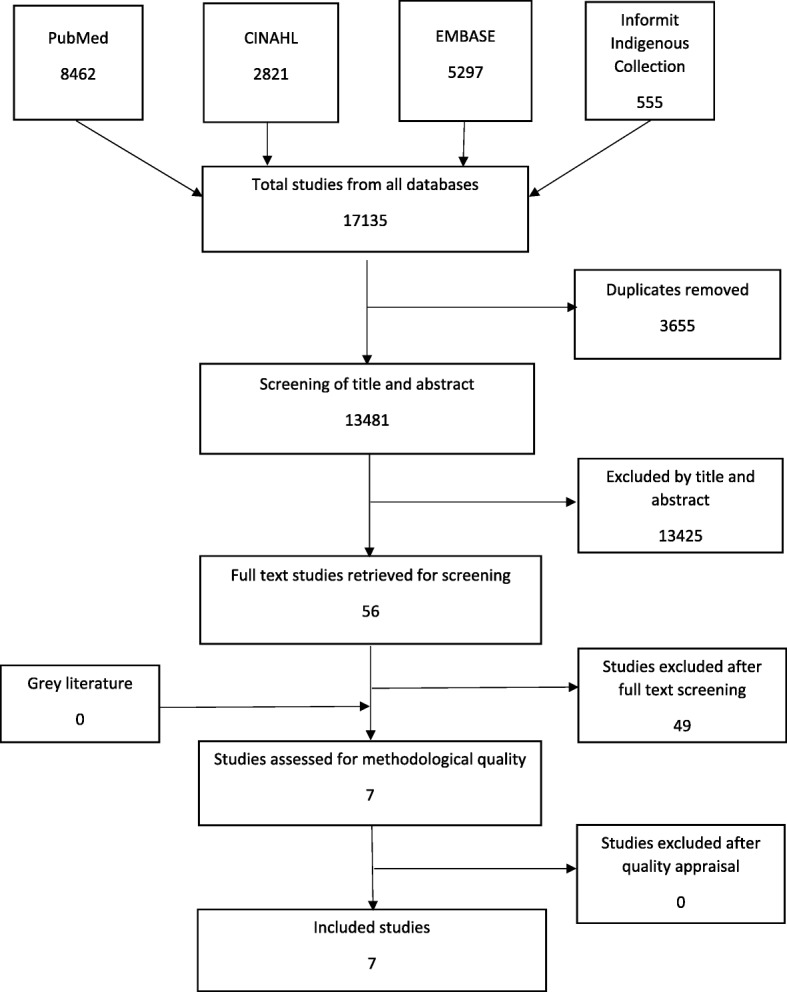
Search Strategy Results

The search of databases found a total of 13,480 publications once duplicates were removed. The full text of 56 papers were retrieved which resulted in seven studies being included in the review. The extensive grey literature search did not retrieve any additional papers. The search results are displayed in Fig. 1.

Of the seven included articles, one was a mixed methods study [26], three involved qualitative methods [33–35] and the other three were narrative opinion papers [27, 28, 36]. Two of the qualitative papers were from the same study [34, 35]. No studies were found that met the inclusion criteria for question 2; implemented strategies to increase utilization of primary health care services by Indigenous men.

### Methodological quality

The methodological quality of the papers was assessed using the Joanna Briggs Institute (JBI) QARI Critical Appraisal Checklist for the Interpretive & Critical Research [32]. The quality assessment of the qualitative papers, and qualitative component of the mixed methods paper that met the inclusion criteria are presented in Table 1. Only Adams et al. mentioned how important “the insider/outsider status of the lead researcher (MJA) was pivotal to the study” [26] (p.33). The other three papers [33–35] failed to address the researchers influence on the study and vice-versa, that is, the role or influence of the researcher on the study and the study’s influence on the researcher, was not critically explored. Hughes 2004 [33] also failed to include evidence that the research had ethics approval.

**Table 1:** Methodological quality of the qualitative studies

	Congruency between philosophical perspective and research methodology.	Congruency between research methodology and research question.	Congruency between research methodology and data collection method.	Congruency between research methodology and representation and analysis of data.	Congruency between research methodology and interpretation of results.	Statement locating the researcher culturally or theoretically.	Is the researchers influence on the research and vice-versa addressed?	Participant’s voices adequately represented.	Evidence of ethics approval and ethical conduct.	Conclusions flow from the analysis or interpretation of the data.
Hughes 2004 [33]	U	Y	Y	Y	Y	N	N	Y	N	Y
Isaacs et al. 2012 [34]	Y	Y	Y	Y	Y	N	N	Y	Y	Y
Isaacs et al. 2013 [35]	Y	Y	Y	Y	Y	N	N	Y	Y	Y
Adams et al. 2013 [26]	Y	Y	Y	Y	Y	Y	N	Y	Y	U

Abbreviations: *Y* yes, *N* no, *U* unclear

The expert opinion pieces [27, 28, 36] that met the inclusion criteria were assessed for quality using the JBI Critical Appraisal checklist for Narrative, Expert Opinion and Text tool. The results of the assessment are displayed in Table 2. The methodological quality assessment tool for expert opinion manuscripts asks if the source of the opinion has standing in the field. After consultation with experts by the lead author, it must be acknowledged that at the time the article was published, Briscoe [27] was considered to have important standing in the field of Indigenous men’s health. In saying this, his role in contemporary research and health policy and practice is less clear. As such, for the purposes of this appraisal the question was answered as ‘unclear’ for Briscoe’s [27] manuscript.

**Table 2:** Methodological quality of the expert opinion manuscripts

	Is the opinion clearly identified?	Does the source of the opinion have standing in the field?	Are the interests of the patients the central focus?	Is the opinion’s basis in logic/ experience clearly argued?	Is the argument development analytical?	Is the reference to the extant literature/ evidence and any congruency with it logically defended?	Is the opinion supported by peers?
Briscoe 2000 [27]	Y	U	Y	Y	Y	Y	Y
Hayman 2000 [28]	Y	Y	Y	Y	Y	Y	Y
Wenitong & Adams 2014 [36]	Y	Y	Y	Y	Y	Y	Y

Abbreviations *Y* yes, *N* no, *U* unclear

It should be noted that the methodological quality can only be judged on what was published in the manuscript and is not necessarily an accurate reflection on the quality of the study.

### Characteristics of included studies

The four manuscripts that included qualitative research methods [26, 33–35] describe three studies. As stipulated in the review protocol manuscripts related to the same study have been combined for the description of study characteristics and results [31].

The basic study characteristics have been summarized in Table 3. All studies included only Indigenous men. Isaacs et al. [34, 35] and Adams et al.’s [26] studies were conducted in Australia, Isaac et al. [34, 35] in Victoria in an urban setting, and Adams et al.’s [26] across urban, rural and remote communities in Queensland and the Northern Territory. Hughes’s [33] study was conducted in Hawaii in both rural and urban settings.

**Table 3:** Characteristics of the qualitative studies

Reference	Study design and objective(s)	Participants	Setting/context	Finding
Hughes 2004 [33]	Qualitative To identify modifiable barriers and to use men’s ideas to develop effective cancer-related programs for Hawaiian men.	Native Hawaiian men aged 22 to 75 participated in four, semi-structured focus groups (N = 54).	Three urban and rural focus group on the island of O’ahu and one group on the island of Hawai’i. All focus group interviews were conducted at community locations not involved in the delivery of health care service.	Study findings suggests that men postpone healthcare services for many reasons, some of which can be addressed through programs.
Isaacs et al. 2012 [34] & Isaacs et al. 2013 [35]	Qualitative Description Describe the perceptions of Aboriginal people and mental health personnel on ways to improve Aboriginal men’s access to mainstream mental health services.	Interviews: 17 Aboriginal male participants (5 Aboriginal mental health clients, 5 community members, 2 cultural advisors, 2 Aboriginal carers of men diagnosed with a mental illness, 1 Koori Hospital Liaison Officer, and 2 social and emotional wellbeing workers. Focus Groups: 3 community mental health team’s members (N = 8, 10, 6)	Victoria, Australia (urban). Interviews: University, Aboriginal Organizations and 3 conducted at the participant’s home. Focus Groups: The teams’ Aboriginal Organization.	Barriers to help seeking by Aboriginal men with mental health problems were identified. Mismatches between mainstream mental health services and the mental health needs of Aboriginal men were identified along with some solutions. Mismatches included barriers to gaining entry, barriers to engaging with services and staffing problems in the service. Potential solutions included building the confidence of men in the services, developing relationships with the community and strengthening the role of the Koori Mental Health Liaison Officers (KMHLOs).
Adams et al. 2013 [26]	Mixed Methods To better understand help-seeking behaviors and reproductive health issues among Aboriginal and Torres Strait Islander men. To report the prevalence of erectile dysfunction, and the possible determinants of erectile dysfunction and prostate health.	Questionnaires: N = 293 Aboriginal and Torres Strait Islander men, aged 18-74. (Includes the interviews and focus group participants). Interviews: 18 men (29-45 years old). Focus Groups: N = 20 in each group (Three men’s groups and one women’s group).	Urban, rural and remote communities from Darwin (urban), Tiwi Islands (remote), Cairns (urban), Yarrabah (remote), Brisbane (urban), Caloundra (rural), and Hervey Bay (rural).	Diabetes, heart disease and high blood pressure frequently reported by the men in the study and high rates of chronic disease coexist with reproductive health problems. Study highlights the low rate of men seeking help for erectile dysfunction. Increases in reported erectile problems prevalence increased with age. Study provides insights to the barriers to seeking help for reproductive disorders and may point ways to improve access to health services.

Isaac et al.’s [34, 35] study focused on improving the accessibility of mainstream mental health services by Indigenous men. Adams et al.’s [26] study focused on Indigenous men accessing reproductive health services, whilst Hughes [33] focused on health seeking behaviors of Indigenous men to inform future effective cancer-related programs. All studies [26, 33–35] identified factors that impact Indigenous men accessing primary health care services.

The findings of the qualitative studies [26, 33–35] have been classified under four organizing themes which relate to health services, attitudes, knowledge and other. The health services theme included the services provided, service settings and the health service staff. The ‘Attitudinal’ theme comprised of the attitudes of Indigenous men and their communities. ‘Knowledge’ related to Indigenous men’s and the community’s knowledge and available information. An additional category for findings that fell outside of these three organizing themes was labelled ‘Other’ (Table 4).

**Table 4:** Factors impacting health seeking behaviors

Study	Health Services	Attitudinal	Knowledge	Other
Hughes 2004 [33]	Distance to services and lack of transport. Lack of traditional healing services. Difficulty getting an appointment. Would like to be able to call a doctor 24-hours a day on a toll-free number for anonymous advice for personal and sensitive health concerns. Lack of specialty services in rural areas. Past experiences of personal interactions with health personnel (positive and negative). Lack of Native Hawaiian health professionals. Physicians need to listen more, have a sense of humor and be more honest.	Fear, shame, embarrassment and distrust. Too embarrassed to seek help for sexual health problems, substance misuse and mental health services. Preventative visits are reassuring. Reluctant to find out something is wrong. Discomfort with some procedures. Less shame if you don’t know the service provider.	Lack of information on services from health institutions and clinics. Participant’s knowledge of the importance of annual health checks.	Financial issues; cost and health insurance coverage. Feeling rushed, ignored or discriminated against because of their insurance coverage. Conflicting priorities. Lack of reminders. Medical bureaucracy. Difficulty finding parking.
Isaacs et al. 2012 [34] & Isaacs et al. 2013 [35]	Having to wait for an appointment. Past negative experiences with health services/ hospitals. Lack of trust with hospitals and health services. Lack of confidentiality. Racial discrimination within services.	Distrust of health services and staff. Shame contacting services. Fear of hospitals and health services. Need to safe-guard their role in society. Peer pressure. Stigma. Fear of being labelled ‘mental’ by community. Perceived need to be ‘strong’.	Difficulty recognizing mental health problems.	Availability of alternative coping strategies; alcohol and other substances.
Adams et al. 2013 [26]	Lack of culturally appropriate health services. Culturally appropriate and gender specific staff required.	Shame, embarrassment and low self-esteem limits ability to talk about health problems. Fear of lack of confidentiality. Stigma about sexual health problems.	Limited education about erectile dysfunction is available.	

The three studies [26, 33–35] found that participants felt there were barriers directly related to the health services and staff. Participants felt services were culturally inappropriate, racially discriminatory, and lacked traditional healing, Indigenous health professionals and gender specific staff. Past experiences with services also influenced the men’s likeliness to access services, with negative experiences reducing their likelihood to return. Isaacs et al.’s [34, 35] study also found that Indigenous men were utilizing alternative coping strategies, including destructive behaviors such as alcohol and other substance use rather than accessing health services.

Distrust was a common theme; distrust of services, their staff or perceived lack of confidentially were factors negatively impacting access. Two studies [33–35] identified difficulty in obtaining an appointment or the long wait times for appointments to be barriers. Hughes et al. [33] found additional barriers to access including conflicting priorities, a lack of reminders for appointments, medical bureaucracy and difficulty finding parking. Beyond this they identified a lack of services, including specialty services in rural areas and the distance and absence of transport limited access to services.

The attitudes of Indigenous men and their communities influenced their help seeking behaviors. Shame, fear and stigma were commonly identified barriers. Shame or embarrassment prevented Indigenous men from talking about their health problems and in some cases from contacting or accessing health services at all. Fear was associated with breaches of confidentiality, fear of hospitals, procedures, receiving bad health news, and fear of being labelled by others. Stigma, in relation to sensitive health issues (mental health and sexual health) was identified as a barrier to health seeking behavior.

Hughes et al.’s [33] study identified barriers related to health insurance coverage and how some men felt discriminated against in relation to this coverage, “eight men commented on how they felt at the doctor’s office, with only two saying they were made to feel comfortable and six saying they felt rushed, ignored or discriminated against because of their insurance coverage”[33] (p. 179). Unfortunately, there was no information regarding the exact type of discrimination experienced by the six men.

Participants from Hughes et al. [33] also found that health preventative checks could be reassuring and that seeing a health provider they did not know can reduce shame. Further, participants in this study identified a potential enabler of a 24-h toll-free number for anonymous advice from a doctor for personal and sensitive health concerns would improve health seeking behaviors. However, the type of doctor (male, female or Indigenous, non-Indigenous) was not mentioned.

The studies [26, 33–35] concluded that additional knowledge would improve service access; not knowing the importance of health checks or being able to identify health problems were factors that reduced primary health care utilization. One study [33] also found that additional information on the health services and clinics would also be beneficial.

## Discussion

It has been well documented that the introduction of western diseases, enforced policies of genocide, assimilation, dispossession and deprivation, have all contributed to the poor health of Indigenous people in many countries [20, 37, 38]. The on-going colonization process continues to be a catalyst towards the poor health and social and emotional wellbeing of Indigenous people [11, 12, 14–16, 18, 19]. Health services play a significant role in maintaining the health and wellbeing of individuals and populations. Their effectiveness is determined by several factors, none more so than their reach. Aboriginal and Torres Strait Islanders are known to utilize health services less often than non-Indigenous Australians, with Aboriginal and Torres Strait Islander men the population with the lowest utilization. The studies reviewed found that there are areas that could be improved to increase the accessibility of health services by Indigenous men. It is important to note that barriers to accessing services and negative experiences reduces the likeliness of patients returning and can lead to unhealthy coping strategies.

Modifications to primary health care services can reduce barriers for Indigenous men accessing care. Interviews with health service users provide valuable information identifying barriers, enablers and identifying potential strategies to improve service utilization. Common themes from the qualitative studies were Indigenous men feeling that services and staff were culturally inappropriate and racially discriminatory. Strategies to improve cultural appropriateness discussed by the research papers and Wenitong et al., include cultural safety/ competency training and the employment and utilization of Indigenous health staff. The re-orientation of health services to suit Indigenous men should be a collaborative process involving local Indigenous men and relevant stakeholders.

Negative attitudes that Indigenous men have about primary health care services impact their utilization. Indigenous men report feeling shame or that it is inappropriateness to discuss health issues, especially for issues such as sexual health and mental health, with a female health professional. Sometimes men want to speak to men and this can be a major barrier for Indigenous men. All expert commentaries (Briscoe, Hayman, Wenitong) discussed the importance of employing gender specific staff, especially for sensitive health issues. Examples of gender specific primary health care services and men’s groups were also discussed as examples of successful strategies for reducing shame and increasing the utilization of primary health care services by Indigenous men. The stigma around some health issues, such as mental health and sexual health is also a barrier to accessing services. The shifting of social norms around such stigmas will take significant time, in the meantime primary health care services should acknowledge the local stigmas that exist and work with local Indigenous men to find ways to make accessing service more appropriate.

There can also be feeling of distrust, particularly around confidentiality within the service. This is likely to be due to past experiences or community rumors. Primary health care services need to be aware of this, ensure all staff are following the strictest protocols for protecting client’s privacy and recognize that some staff that have relationships with clients may be inappropriate to be involved in the delivery of their care, data entry and handling of their information. Increasing the familiarity of Indigenous men with the service and staff may also help reduce feels of distrust. Participants from the Isaacs et al. study suggested group visits to services so that men become familiar with the services and importantly the staff [34].

There is a lack of information about health services available at local primary health care services and understanding the benefits of utilizing them. It was found that men feel that they do not have enough information on when to go and for what. Investing in education for men in these areas to help Indigenous men recognize health issues that require attention, the importance of annual health checks and where to go for what may increase the utilization of primary health care services by Indigenous men.

There are limitations to this study. The search for peer-reviewed literature was limited to four databases and did not include a search for online theses. The search for grey literature was restricted to Australia, likewise only expert opinions from Australian Aboriginal and/or Torres Strait Islander health experts were included. Although the review included literature from New Zealand, Canada and America, the purpose of the review is to inform primary health care services within the Australian context. The researchers being situated in Australia also reduced the ability to capture grey literature from other countries. Expert opinions published by non-Indigenous authors were not included, this decision was made to privilege Aboriginal and Torres Strait Islander voices and to highlight the importance of Indigenous leadership to improve Indigenous health [39].

There is a current lack of published research in this area. The inclusion criteria for this review only included papers where men were majority of the participants (over 50%). This included publications that included some male voices within broader papers of which most of the responses were from women. The research included [26, 33–35] in this literature review has been conducted across multiple sites in Australia and in Hawai’i, throughout urban, rural and remote settings. One of the studies was specific to mental health services and another was specific to sexual health, therefore the results may not be generalizable to primary health care services.

## Conclusions

The literature search found limited published research meeting the inclusion criteria. The factors impacting primary health care utilization described by Indigenous male participants were found within three studies [26, 33–35]. In addition, the search found three expert opinion papers [27, 28, 36] but no published literature was found describing studies that have implemented and accessed the effectiveness of strategies to improve primary health care utilization by Indigenous men.

Of the studies identified, the factors impacting primary health care utilization shared many similarities. Indigenous men described factors impacting utilization which were categorized into the following organizing themes; health services, attitudes of Indigenous men and knowledge. Many of these findings echoed the sentiments of the three expert opinion papers [27, 28, 36].

The studies reviewed found that there are areas that could be improved to increase the accessibility of health services by Indigenous men. There were common themes found by the research including; the need for culturally appropriate services and staff, increased knowledge, distrust and fear of health services, shame and stigma especially around sensitive health issues. The evidence also highlighted the need for gender specific services and male health providers. Even though there is limited research that explores the barriers for health service utilization by Indigenous men, the evidence available supports the anecdotal evidence and expert opinion. The barriers and enablers identified in this review should be used to inform the development of new strategies to improve the utilization of health services by Indigenous men.

Currently there is insufficient data available on the utilization of primary health care services by Indigenous men. It is widely acknowledged that Indigenous men are underutilizing services, however, unless this information is shared we will not be able to track progress in the improvements of service utilization. Future research should focus on evaluating the implementation of men specific utilization strategies. It is through such evidence-based research that subsequent policies and programs can be made and implemented to improve Indigenous men’s health.

## Abbreviations

JBI: Joanna Briggs Institute
JBI-NOTARI: Joanna Briggs Institute Narrative, Opinion and Text Assessment and Review Instrument
JBI-QARI: Joanna Briggs Institute Qualitative Assessment and Review Instrument
